# Developmental hypomyelination in Wolfram syndrome: new insights from neuroimaging and gene expression analyses

**DOI:** 10.1186/s13023-019-1260-9

**Published:** 2019-12-03

**Authors:** Amjad Samara, Rachel Rahn, Olga Neyman, Ki Yun Park, Ahmad Samara, Bess Marshall, Joseph Dougherty, Tamara Hershey

**Affiliations:** 1 Department of Psychiatry, Washington University School of Medicine, 4525 Scott Avenue, St. Louis, MO 63110 USA; 2 Mallinckrodt Institute of Radiology, Washington University School of Medicine, St. Louis, MO 63110 USA; 3 Department of Genetics, Washington University Medical School, St. Louis, MO 63110 USA; 4 Faculty of Medicine and Health Sciences, An-Najah National University, Nablus, Palestine; 5 Department of Pediatrics, Washington University Medical School, St. Louis, MO 63110 USA; 6 Department of Neurology, Washington University School of Medicine, St. Louis, MO 63110 USA

**Keywords:** *WFS1*, endoplasmic reticulum stress, Unfolded protein response, Neuroimaging, Hypomyelination, Neurodevelopment, Neurodegeneration

## Abstract

Wolfram syndrome is a rare multisystem disorder caused by mutations in *WFS1* or *CISD2* genes leading to brain structural abnormalities and neurological symptoms. These abnormalities appear in early stages of the disease. The pathogenesis of Wolfram syndrome involves abnormalities in the endoplasmic reticulum (ER) and mitochondrial dynamics, which are common features in several other neurodegenerative disorders. Mutations in *WFS1* are responsible for the majority of Wolfram syndrome cases. *WFS1* encodes for an endoplasmic reticulum (ER) protein, wolframin. It is proposed that wolframin deficiency triggers the unfolded protein response (UPR) pathway resulting in an increased ER stress-mediated neuronal loss. Recent neuroimaging studies showed marked alteration in early brain development, primarily characterized by abnormal white matter myelination. Interestingly, ER stress and the UPR pathway are implicated in the pathogenesis of some inherited myelin disorders like Pelizaeus-Merzbacher disease, and Vanishing White Matter disease. In addition, exploratory gene-expression network-based analyses suggest that *WFS1* expression occurs preferentially in oligodendrocytes during early brain development. Therefore, we propose that Wolfram syndrome could belong to a category of neurodevelopmental disorders characterized by ER stress-mediated myelination impairment. Further studies of myelination and oligodendrocyte function in Wolfram syndrome could provide new insights into the underlying mechanisms of the Wolfram syndrome-associated brain changes and identify potential connections between neurodevelopmental disorders and neurodegeneration.

## Background

Wolfram syndrome (OMIM #222300; previously known as DIDMOAD) is a rare (1 in 500,000 to 1,000,000), autosomal recessive disease initially described as a combination of early-onset diabetes mellitus, progressive optic nerve atrophy, diabetes insipidus and sensorineural hearing loss [1]. About two-thirds of the patients diagnosed with Wolfram syndrome will ultimately develop all four of the clinical conditions. Other features of Wolfram syndrome include bladder and bowel dysfunction, temperature dysregulation, gait abnormalities, and loss of the senses of smell and taste. Wolfram syndrome symptoms have a negative impact on individuals’ daily function and quality of life [1, 2]. Symptoms of Wolfram syndrome usually start in the first two decades of life and progress over years [3]. Currently, there is no treatment to stop the progression of the disease, and many symptoms can be life-threatening [1, 3].

Two genetically distinct variants have been identified, type-1 and type-2 Wolfram syndrome, which result from mutations in the *WFS1* and *CISD2*, respectively [4, 5]. Type-1 Wolfram syndrome is much more common [4, 6]. Type-2 Wolfram syndrome presents with the four primary features of type 1 Wolfram syndrome, with the additional features of gastrointestinal ulcers, platelet dysfunction, and absence of diabetes insipidus [6]. Interestingly, mutations in the *WFS1* are not implicated only in the pathogenesis of classic Wolfram syndrome, but are also involved in the development of other *WFS1*-related disorders such as DFNA6/14/38 (OMIM #600965) non-syndromic low-frequency sensorineural hearing loss, non-syndromic autosomal-dominant diabetes, and Wolfram-like syndrome (OMIM #614296) [7–9]. Syndromes that have mutations in *WFS1* but do not meet the diagnostic criteria of Wolfram syndrome (diabetes mellitus and optic atrophy) are referred to as *WFS1*-related disorders. *WFS1* encodes for a putative endoplasmic reticulum (ER) protein called wolframin [10]. Since its discovery, growing evidence suggests that wolframin plays a crucial role in the regulation of ER stress and Ca^2+^ homeostasis and that its deficiency triggers proapoptotic pathways leading to cellular loss [11–13].

Wolfram syndrome can be associated with significant neurological and psychiatric complications. Patients with Wolfram syndrome experience a wide range of neurological complications including cerebellar ataxia (most common), gait and balance abnormalities, and as the disease progresses, difficulty swallowing, nystagmus, memory loss, speech difficulties, seizures, and personality changes [14, 15]. Neurological manifestations were thought to appear at later stages of the disease, but recent evidence indicates some of these neurological abnormalities are present even at young ages [16, 17]. Besides these neurological complications, several psychiatric manifestations, including anxiety and depression, can also occur early in the course of Wolfram syndrome [16, 18, 19]. The molecular pathophysiology underlying the neurological and psychiatric manifestations of Wolfram syndrome is not well understood. In general, the field is hampered by a lack of a viable conceptual framework and thus is missing hypothesis-driven experimentation focused on the central nervous system impact of *WFS1* mutations [20–22].

In this review, we attempt to summarize our current understanding of the Wolfram syndrome-related structural and functional brain alterations and to provide insights from new neuroimaging and *WFS1* expression analyses across age and cell types. We highlight similarities and differences compared to other neurodevelopmental and white matter diseases of childhood. Together, this information suggests that Wolfram syndrome could belong to a category of neurodevelopmental disorders characterized by ER stress-mediated myelination impairment. However, studies investigating the roles of *WFS1* in myelinating oligodendrocytes are limited, and further histopathological and molecular genetic studies are necessary to confirm this hypothesis.

## Molecular mechanisms in Wolfram syndrome

### *WFS-1/CISD-2* genes and Wolframin protein

The pathogenesis of Wolfram syndrome is attributed to genetic mutations in two genetic loci on chromosome 4 (*WFS1* and *CISD2*- also known as *ZCD2* gene or *WFS2* gene) [4, 5]. *WFS1* encodes an endoplasmic reticulum (ER)-associated transmembrane glycoprotein called wolframin. Wolframin seems to play a role in the regulation of cellular and ER Ca^2+^ homeostasis, and to contribute to quality control systems for protein folding and regulation of the ER stress response [11–13]. Loss of function mutations of wolframin triggers a cascade of ER and mitochondrial dysfunction that ultimately leads to apoptosis and cellular death. On the other hand, *CISD2* encodes for an ER intermembrane small protein (ERIS), a protein expressed on the mitochondria-associated ER membranes (MAMs). Mutations in *CISD2* alter Ca^2+^ flux between ER and mitochondria, disrupting organelle function, and leading to autophagy and cell death like that seen in several other neurodegenerative diseases.

**Fig. 1:**
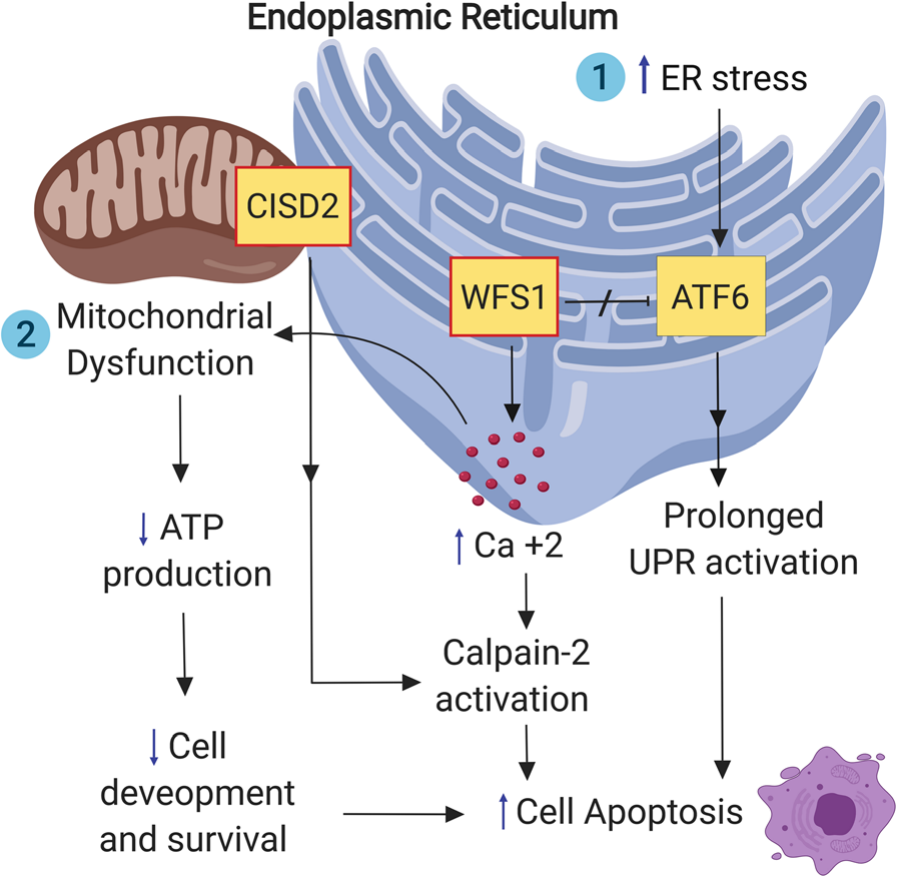
A schematic representation of the endoplasmic reticulum and mitochondrial molecular changes in Wolfram syndrome (the red box indicate a deficiency of this protein). ER: endoplasmic reticulum; ATF6: Activating transcription factor 6; UPR: unfolded protein response; WFS1: wolframin protein; CISD2: CISD2 protein product, ERIS

Different mutations in *WFS1* likely result in different disease phenotypes, but genotype-phenotype relationships are not yet fully understood [3, 23]. Moreover, a novel *CISD2* mutation was discovered recently in a patient with the Wolfram syndrome type 1 classical phenotype, suggesting that type 1 and type 2 Wolfram syndrome could be viewed as a continuous clinical spectrum with overlapping phenotypes, providing a clue that the protein products of *WFS1* and *CISD2* may reside in the same molecular pathway [24]. A summary of the molecular pathways involved in Wolfram syndrome is shown in Fig. 1.

### Role of WFS1/CISD2 in ER stress and unfolded protein response (UPR)

Some authors have considered Wolfram syndrome to be a monogenic prototype of human ER disease and the best human disease model for investigating biomarkers and therapeutics associated with ER health [25]. Disturbances in Ca^2+^ homeostasis and the buildup of misfolded proteins in the ER leads to a condition called ER stress, which has been implicated in the pathogenesis of several neurodegenerative diseases [26, 27]. ER stress triggers an adaptive intracellular pathway, called the unfolded protein response (UPR), which attempts to restore ER homeostasis, by attenuation of general protein translation and increase of the ER capacity of protein folding [28]. However, in cases of chronic and unresolved ER stress, the UPR initiates proapoptotic pathways leading to cell death [29]. Given its localization in the ER, wolframin seems to play a crucial role in ER Ca^2+^ homeostasis, as well as regulation of the ER stress and the UPR, and mutations in *WFS1* have been shown to induce ER stress-mediated neuronal loss [10, 22, 25, 30]. On the other hand, *CISD2* seems to play a less critical role in ER stress pathways, and some scientists suggest that type 2 Wolfram syndrome is actually a mitochondrial disease rather an ER stress-mediated condition [31].

### Mitochondrial dysfunction in Wolfram syndrome

It has long been appreciated that several neurological and psychiatric manifestations in Wolfram syndrome resemble those observed in mitochondrial disorders [32]. Therefore, some authors have suggested that mitochondrial dysfunction is the primary underlying cause of neuronal cell loss in Wolfram syndrome [31, 33, 34]. Specifically, Cagalinec et al. showed that wolframin deficiency results in delayed neuronal development due to pervasive alteration in mitochondrial dynamics like inhibited mitochondrial trafficking and fusion, as well as increased mitophagy – i.e., auto-degradation of the mitochondria [34]. The authors also demonstrated that the alterations in mitochondrial function result from disturbances in cytosolic Ca^2+^ concentrations or could be a direct result of increased ER stress. The communication between ER and mitochondria is essential for cellular Ca^2+^ homeostasis and disruptions of this communication has been implicated in other neurodegenerative conditions [35]. These observations are not surprising since ERIS and multiple UPR effector proteins are indeed located in the mitochondria-associated membranes (MAMs). Taken together, these findings strongly suggest a potential interaction between ER homeostasis and mitochondrial dynamics [35–37].

## Brain histopathology in Wolfram syndrome

**Fig. 2:**
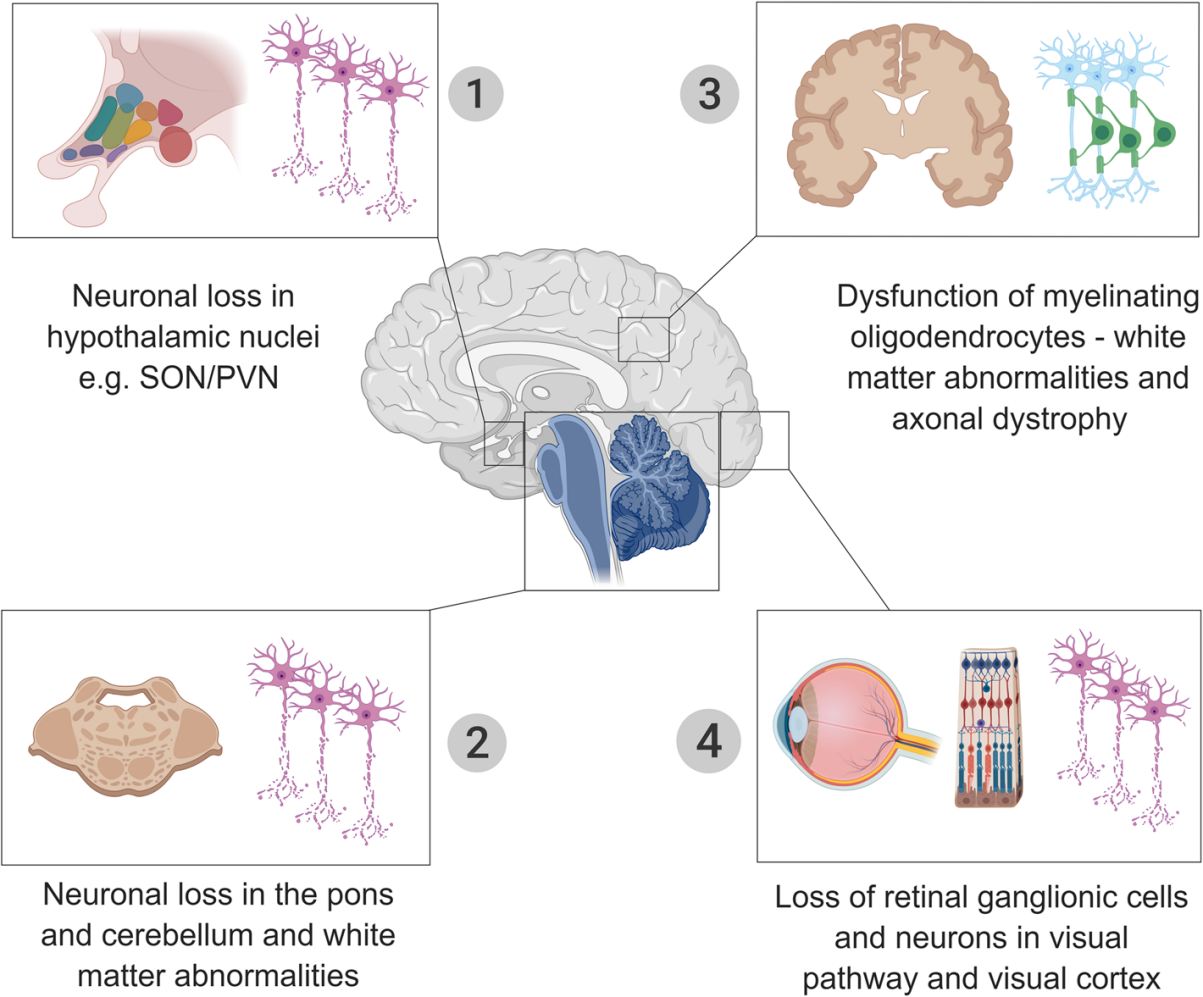
Brain structures and tissues most prominently affected in Wolfram syndrome. SON: supraoptic nucleus; PVN: paraventricular nucleus

Neuropathological studies provide a critical step towards identifying brain regions and structures involved in Wolfram syndrome. A handful of postmortem brain histopathological case studies have been reported (Table 1) [38–41]. However, the reported cases vary in age, the cause of death, and in the scope of methods and tissues examined. Consistently, the most affected brain regions in Wolfram syndrome are the sensory pathways, the brainstem, the cerebellum and the hypothalamus (Fig. 2) [38–41]. In the visual system, the optic nerves appear grossly atrophic and microscopic examination reveals loss of retinal ganglion neurons and myelinated axons throughout the visual pathways with relative preservation of the visual cortex [38–41]. Within the auditory pathway, studies have found loss of the organ of Corti (the functional unit of the inner ear) in the basal turns of the cochlea, fibers in the cochlear nerve, and neurons in the cochlear nuclei and inferior colliculus [39, 40]. Within the olfactory pathway, atrophy of the olfactory bulb and tract has also been reported [39]. The brainstem and cerebellum are grossly smaller in Wolfram syndrome. Microscopic examination finds moderate neuronal loss and gliosis in almost all brainstem nuclei (pontine nuclei, raphe nuclei, inferior olivary nuclei, medial vestibular nucleus, medullary and pontine reticular formation, vagus dorsal nuclei, ambiguous nuclei) [39–41]. In the cerebellum, microscopic evidence of neuronal loss in the dentate nuclei and reduction of Purkinje cells is variably reported and has been an inconsistent finding in these case studies [39–41]. The hypothalamus exhibits significant gliosis and severe loss of magnocellular neurons in the supraoptic and paraventricular nuclei (a group of neurons that project to the posterior pituitary and are responsible for the release of oxytocin and vasopressin) [39–41]. Other brain structures are affected to a lesser extent. For example, the thalamus has been reported to have mild neuronal loss and gliosis in the anterior and dorsomedial nuclei [39]. Other less frequently reported findings include mild axonal damage in the calcarine cortex, mild motor neuron loss and gliosis in the spinal cord, and loss of pigment, neuronal loss, and gliosis in the substantia nigra [38, 39].

**Table 1:** Summary of the histopathological findings in Wolfram syndrome patients

Reference	Patient	Clinical Findings	Histopathological findings
Carson et al. 1977	15 yo M	DM, DI, OA, osteopetrosis, and obstructive uropathy.	**-** Atrophy of the pons, optic nerves, chiasm, and tracts, pale SN, and small posterior pituitary **-** Axonal destruction and loss of myelin in optic nerves, chiasm, and tracts, CST and transverse pontine fibers. Loss of neurons in the PVN and SON.
Carson et al. 1977	21 yo F	CHD and renal anomalies, vision loss, DM, DI, and osteoporosis	**-** Atrophy of optic nerves, chiasm, and tracts, pallor of the OR, pale SN, absent posterior pituitary, atrophy of pons and cerebellum **-** Severe axonal destruction and demyelination in optic nerves, loss of neurons in the PVN and SON, gliosis/atrophy of the superior olivary nucleus, scattered regions of demyelination.
Hilson et al. 2009	24 yo M	DM, DI, vision loss, Hashimoto thyroiditis, nystagmus, SNHL, slurred dysarthria, gait abnormality	- marked neuronal loss in the PVN and SON, pontine base, inferior olivary nucleus, retinal ganglion, myelinated axons in the optic nerves, optic chiasm, lateral geniculate nucleus, and loss of organ of Corti in the basal turn of the cochlea
Genis et al. 1997	37 yo F	DM, anosmia, vision and high-frequency hearing loss, cerebellar dysfunction, clonus, memory loss, dysarthria, postural tremor, and urinary abnormalities	- Atrophy of olfactory bulbs, tracts, optic nerves, chiasm, loss of neurons in LGN, superior colliculus, cochlear nerve and nuclei, inferior colliculus, and OPCT atrophy, demyelination of the pyramidal tracts, neuronal loss of the PVN and SON, and motor neurons in the spinal cord
Shannon et al. 1999	38 yo F	DM, DI, vision loss, SNHL, hyporeflexia, urinary abnormalities, paresthesias, memory impairment, anosmia, strabismus, nystagmus	- Degeneration of optic nerves and tracts, loss of neurons in LGN, basis pontis, PVN and SON, widespread axonal dystrophy in pontocerebellar tracts, OR, hippocampal fornices, and deep cerebral white matter

*DM* diabetes mellitus, *DI* diabetes insipidus, *OA* optic atrophy, *SN* substantia nigra, *PVN* paraventricular nucleus of hypothalamus, *SON* supraoptic nucleus, *CST* corticospinal tract, *CHD* Congenital heart disease, *OR* optic radiation, *SNHL* sensory neural hearing loss, *LGN* lateral geniculate nucleus, *OPCT* olivopontocerebellar tract

From all these case studies, it might be said that there are two distinct histopathological abnormalities associated with Wolfram syndrome: neuronal loss and gliosis in subcortical and limited cortical gray matter, and patchy demyelination and axonal degeneration in several white matter tracts, e.g., optic radiation, pontocerebellar and corticopontine tracts, hippocampal forncies, and the deep cerebral white matter [38–41]. It was suggested that the axonal pathology is an independent process and sometimes more striking than the neuronal loss, which indicates that these could be independent pathological processes [41]. Moreover, we have suggested that, if the cases are arranged according to patient’s age and the severity of the disease, one might conclude that the evolution of the neuropathological alterations follows a specific pattern from restricted myelin and axonal loss to widespread myelin, axonal and neuronal loss [42]. This information could support our hypothesis that abnormal myelination and axonal pathology might precede the neuronal loss. Of note, these conclusions need to be viewed with caution since these histopathologic studies were all published before the era of genetic confirmation for Wolfram syndrome.

## Quantitative neuroimaging in Wolfram syndrome

*In vivo* brain imaging provides a useful tool to assess the histopathological abnormalities in various neurological disorders across time, and without need for postmortem tissue. In Wolfram syndrome, several brain MRI abnormalities are readily detectable by eye [15, 43]. However, until recently, brain imaging findings were exclusively studied in the relatively late stage of the disease, and as case studies without quantification of the findings or comparison with control groups [15, 43]. In adults, the classical neuro-radiological manifestations of Wolfram syndrome include marked atrophy of the brainstem, diffuse cerebellar gray and white matter atrophy, thinning of the middle cerebellar peduncle, absent posterior pituitary T1 bright spot (indicating posterior pituitary degeneration), and optic nerve and optic tract atrophy [15, 43, 44]. Less frequently, MR images show signs indicative of diffuse mild cerebral atrophy, periventricular white matter and ventral pons T2-weighted and fluid attenuation inversion recovery (FLAIR) signal intensity changes, empty sella, and abnormal T2-weighted signal in the substantia nigra [40, 45–47]. Most of these MRI findings were also observed in children with Wolfram syndrome, and as early as the immediate postnatal period in one case of congenital diabetes insipidus [48]. Another remarkable finding in Wolfram syndrome is the discrepancy between the radiological and neurological manifestations in some instances, i.e., marked radiological changes with no or minimal neurological dysfunction [43].

**Fig. 3:**
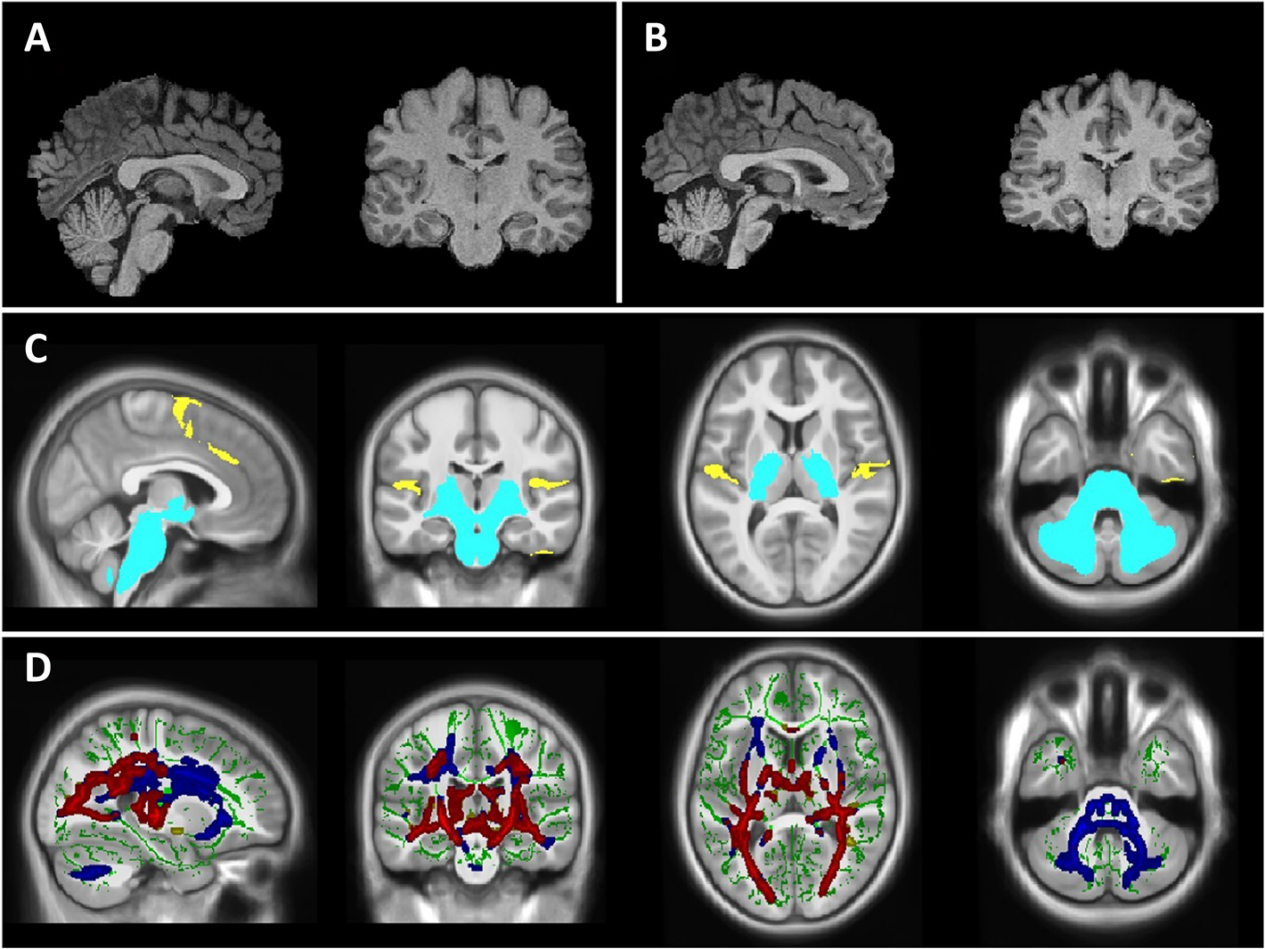
**a**) Sagittal and coronal view of a healthy young adult brain. **b**) Sagittal and coronal view of a young adult brain with Wolfram syndrome. **c**) Significant volumetric differences between Wolfram syndrome and controls, controlling for whole brain volume. Regions that are smaller in Wolfram syndrome are in light-blue, while regions that are larger are in yellow. **d**) White matter microstructure alterations in Wolfram syndrome as measured by diffusion tensor imaging. *Green*: white matter skeleton created by tract-based spatial statistics skeletonization step; *Blue*: white matter tracts with greater radial diffusivity in Wolfram syndrome; *Yellow*: lower fractional anisotropy; *Red*: white matter tracts with overlap of greater radial diffusivity and lower fractional anisotropy is shown in red

The most comprehensive attempt to characterize the structural neuroimaging phenotype in Wolfram syndrome patients, at a relatively early stage, was conducted by our group [16, 42, 49]. Our goal was to quantify regional brain volume and microstructural abnormalities associated with Wolfram syndrome. A summary of these structural neuroimaging findings is shown in Table 2 and Fig. 3. In brief, the intracranial and whole brain volumes, brainstem, cerebellar white and grey matter volumes were lower in Wolfram syndrome when compared to controls (using both region of interest (ROI) and voxel-wise analysis approaches) [16]. The thalamus and pallidum also showed a mildly lower volume but no differences in the volume of striatal structures (putamen, caudate, and nucleus accumbens), hippocampus and corpus callosum [16, 49]. Brainstem volumes were reduced in all segments (midbrain, pons, and medulla) but the difference was most striking in the pons [16, 49]. Reduced cortical thickness was a less prominent finding with the pre-central, lingual, and middle frontal regions mostly affected [16]. White matter microstructure was also examined using diffusion tensor imaging (DTI). Wolfram syndrome patients had significantly lower fractional anisotropy (FA) and higher radial diffusivity (RD) in widespread white matter tracts (optic radiation, middle cerebellar peduncle, inferior fronto-occipital fasciculus, and acoustic radiation) compared to age-equivalent controls [49]. FA is a highly sensitive measure of overall WM microstructural integrity [50, 51] and RD measures water diffusion perpendicular to the principal axonal axis, which is used as a surrogate marker for myelination [51]. The combination of higher RD and lower FA observed in the Wolfram group could indicate impaired myelination in these patients.

**Table 2:** Summary of the quantitative neuroimaging findings in Wolfram syndrome patients

Reference	Neuroimaging findings	Analysis type
Brain volumes		
Hershey et al. 2012 ^a^	- ↓ Intracranial and whole brain volume	
	- ↓ brainstem (midbrain, pons, and medulla) volumes; most striking in pons	ROI
	- ↓ GM volume in the cerebellum.	VBM
	- ↓ WM volume in the cerebellum, brain stem, and subcortex	VBM
Lugar et al. 2016 ^a^	- ↓ total cortical WM and total subcortical GM volumes	
	- ↓ volume in ventral and dorsal pons, midbrain, medulla, cerebellar WM and GM, thalamus and pallidum	ROI
	- ↑ amygdala volume	ROI
Lugar et al. 2019 ^a^	- WM volumes were stable (OR) or decreasing (brainstem and ventral pons)	ROI, VBM
	- GM volumes were decreasing in thalamus and cerebellar cortex	ROI, VBM
Cortical thickness/cortical volumes		
Hershey et al. 2012	- ↓ cortical thickness in pre-central region, lingual region, and two rostral middle frontal region	
Lugar et al. 2016	- ↓ cortical thickness and cortical volume in primary and secondary visual cortex; higher cortical volume and surface area in the primary and secondary auditory cortex	ROI
	- ↓ peri-calcarine region surface area, thickness, and volume; ↓ volume in parahippocampal region; ↑ temporal lobe regions surface area and volume; ↑ rostral middle frontal volume and thickness	QDEC vertex-wise cortical metrics
Diffusion MRI and white matter integrity		
Hershey et al. 2012	- ↓ FA in the cerebellum and OR + ↓ MD in the cerebellum	ROI
	- Widespread ↓ FA and AD, mostly in the brainstem, cerebellum and OR	TBSS
Lugar et al. 2016	- ↓ FA and ↑ RD in OR, MCP, IFOF, and AR; and ↑ AD in OR	Tractography
	- ↓ FA (CST, IFOF, OR, CC); ↑ RD (MCP, CST, ILF, OR, SLF); ↑ AD (MCP, IFOF, ILF, ALIC)	TBSS

^a^ Participants enrolled in these three studies overlap (Hershey et al. 2012: 11 Wolfram patient/ 54 type 1 diabetes and healthy controls; Lugar et al. 2016: 21 Wolfram patients/ 50 type 1 diabetes and healthy controls; Lugar et al. 2019: 29 Wolfram patients/ 52 type 1 diabetes and healthy controls)

*GM* gray matter, *WM* white matter, *ROI* region-of-interest, *VBM* voxel-based morphometry, *QDEC* Query, Design, Estimate, Contrast, *FA* Fractional anisotropy, *OR* optic radiation, *MD* mean diffusivity, *AD* axial diffusivity, *RD* radial diffusivity, *TBSS* tract-based spatial statistics, *MCP* middle cerebellar peduncle, *IFOF* inferior fronto-occipital fasciculus, *AR* acoustic radiation, *CC* corpus callosum, *CST* corticospinal tract, *ILF* inferior longitudinal fasciculus, *SLF* superior longitudinal fasciculus, *ALIC* anterior limb of internal capsule

Recently, we also examined changes in brain volumes over time in Wolfram syndrome compared to controls. Using voxel-wise morphometric longitudinal analyses, we found that specific white and gray matter volumes were affected during development in Wolfram syndrome [42]. Over time and age, white matter volumes tend to increase in controls, reflecting increased myelin, and gray matter volumes tend to be stable (subcortex) or decrease (cortex). In contrast, patients with Wolfram syndrome had stable (in optic radiations) or decreasing (in brainstem, ventral pons) white matter volumes and more sharply decreasing volumes in the thalamus and cerebellar cortex. These findings are consistent with stalled or deficient myelination during development in Wolfram syndrome and subsequent or simultaneous excessive loss of axons and cell bodies over time [42]. Importantly, it seems unlikely that the brain abnormalities described in Wolfram syndrome patients are the consequence of diabetic complications. In the previous studies, Wolfram syndrome patients were compared to healthy and type 1 diabetic controls with comparable glycemic profiles, yet Wolfram syndrome patients were qualitatively different from both control groups [16, 42, 49].

In contrast to these structural studies, functional neuroimaging studies in Wolfram syndrome are relatively lacking. To date, only one study has quantitatively investigated functional brain changes in Wolfram syndrome. This study showed regional differences in glucose uptake measured by PET-CT scan in several brain regions, most notably the occipital lobe and cerebellum [52]. The authors of this study suggested that functional alterations in Wolfram syndrome may precede detectable structural changes.

## Neurodevelopment and Wolfram syndrome

Evidence that *WFS1* could play a vital role in brain development comes from several clinical, neuroimaging, and genetic observations, yet the role that *WFS1* plays in the healthy developing brain is not completely understood. Clinically, manifestations of Wolfram syndrome have been reported as early as the intrauterine and early postnatal life in a child with neonatal-onset congenital diabetes insipidus [48, 53]. Furthermore, Wolfram syndrome has even been associated with several congenital anomalies including orbital bone and eye globe hypoplasia [53], neural tube defects like spina bifida [54], and potentially, microcephaly [16, 40]. In the endocrine system, the role of *WFS1* in organ embryogenesis has been documented in the pancreas, specifically showing a lower number of pancreatic islets in *wfs-1* deficient mice when compared to heterozygous and wild-type mice [55]. Neuroimaging studies have also shown that Wolfram syndrome has a pronounced impact on early brain development [16]. For example, Wolfram syndrome has also been associated with other congenital brain anomalies like thinning or agenesis of the corpus callosum, congenital hypoplasia of the optic nerve, and absent pituitary stalk [48].

Molecular genetic studies have shown that wolframin deficiency may impair early neuronal survival and delay neuronal development [34]. *WFS1* is expressed during brain development, and the downstream molecular pathways affected by wolframin deficiency (e.g., UPR and mitochondrial dynamics) also play a crucial role in early brain development, e.g., neurogenesis, neuronal migration, and myelination [34, 56, 57]. Although the UPR is known to be activated during normal developmental myelination, *WFS1*-dependent pathways in oligodendrocytes and astrocytes have never been investigated. *WFS1* expression and function may, therefore, be different during development compared to adult life and understanding the patterns of gene expression in early life could provide relevant information about the disease pathogenesis. Taken together, Wolfram syndrome could be considered a neurodevelopmental condition with neurodegeneration occurring in later stages of the disease. Further studies are required to confirm the role of *WFS1* expression in early brain development and how wolframin deficiency could influence neuronal cell differentiation and maturation.

## Oligodendrocytes and myelination

The exact role of *WFS1* in astrocyte and oligodendrocyte function and the effects of wolframin deficiency in these cell types are still not investigated. Experiments in all studies that investigated the molecular mechanisms of Wolfram syndrome were conducted in either neurons, fiberoblasts, or pancreatic cells but not in glial cells or oligodendrocytes As we explained above, recent neuroimaging studies suggest that abnormal myelin development is a primary neuropathological feature of Wolfram syndrome observed from a young age [49]. One possible explanation is that wolframin deficiency alters myelinating oligodendrocytes’ function and interferes with myelin development. Another explanation could be that ER stress triggers oligodendrocyte death and facilitates myelin degeneration, as it does in Pelizaeus-Merzbacher (PMD) and Vanishing White Matter Diseases (VWMD) [49, 58, 59]. Moreover, in blood samples from patients with Woflram syndrome, greater levels of cleaved myelin basic protein (MBP), a major component of myelin sheath, correlated with the severity of clinical symptoms [49]. Although these observations support our hypothesis, it is possible that the abnormal myelination could be related to underlying axonal pathology since the preservation of the myelin sheath requires the support of the associated axons [60].

In the rodent brain, *WFS1* is expressed in several central nervous system (CNS) regions including cerebral and cerebellar cortex, amygdala, CA1 field of the hippocampus, hypothalamus, basal ganglia, and several brainstem nuclei [10, 20, 61]. Moreover, *WFS1* is also ubiquitously expressed in retinal ganglion cells and optic nerve glial cells [62, 63]. However, expression patterns of *WFS1* differ in regional and temporal relations in postnatal development [61]. Kawano et al. suggested that *WFS1* could have functional significance in the development and maintenance of neurons in the hypothalamic nuclei, the auditory system including the cochlea, and the cerebellum. It is intriguing that *WFS1* expression is observed in widespread CNS regions while the neuronal loss is only observed in specific structures like cerebellum, optic pathway, and brainstem. It is tempting to speculate that the neurons less affected by *WFS1* mutations could have, a yet unknown, functionally-related protein or pathway to compensate for wolframin deficiency and could explain the preferential vulnerability in certain brain regions. Taken together, *WFS1* expression patterns in various brain structures could inform knowledge relevant to the neurological and psychiatric symptoms observed in Wolfram syndrome.

Oligodendrocytes (the myelinating cells of the CNS) play a crucial role in the development and maintenance of axonal integrity, providing metabolic support through the myelin sheath [64]. Oligodendrocytes produce massive amounts of plasma membranes and transmembrane proteins during the myelination process making them especially vulnerable to secretory pathway disruptions [65]. Previous studies have shown that UPR activation in actively myelinating oligodendrocytes triggers apoptosis and cell death [66]. It is possible that wolframin deficiency in actively myelinating oligodendrocytes leads to activation of the UPR, resulting in oligodendrocyte death and abnormal myelination. Unfortunately, the roles of *WFS1* and wolframin protein in the oligodendrocytes have never been investigated. We hypothesize that *WFS1* plays an essential role in oligodendrocyte function. To begin to investigate the hypothesis that *WFS1* plays an essential role in oligodendrocyte function, we performed an exploratory analysis of *WFS1* expression in development and across specific cell types.

### *WFS1* gene expression

**Fig. 4:**
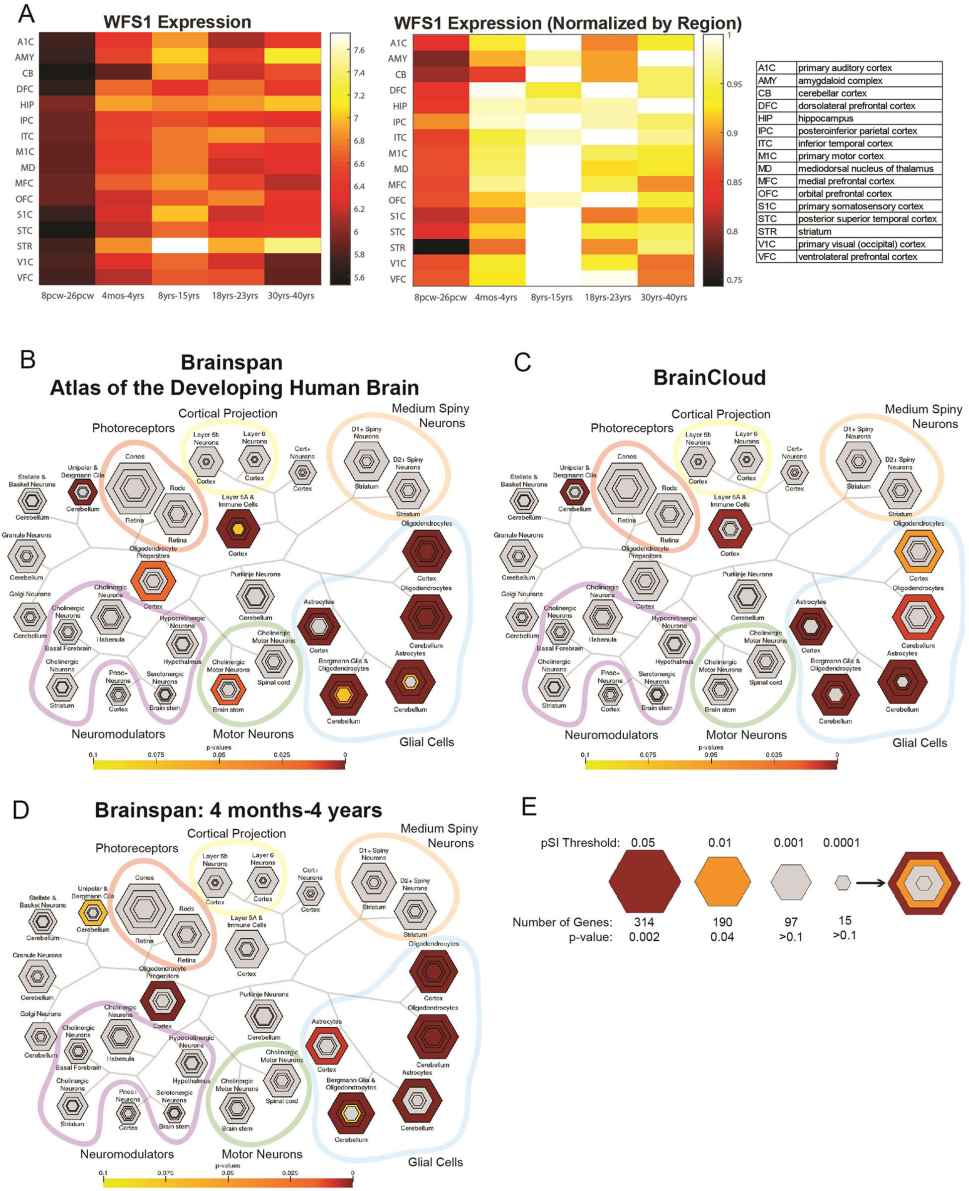
Temporal expression of *WFS1* and cell type-specific expression of *WFS1*-related genes. **a**) Left: Mean *WFS1* spatiotemporal expression (RPKM, or reads per kilobase per million) in 16 brain regions and 5 developmental time periods from the BrainSpan database (8–26 post-conception weeks (pcw), 4 months-4 years, 8 years–15 years, 18 years–23 years, and 30 years–40 years). Right: Mean WFS1 spatiotemporal expression normalized to each brain region’s expression across time. **b**) Cell-type specific expression in the human brain of *WFS1*-related genes. Gene list derived from BrainSpan database brains 8pcw-40 yrs. **c**) Cell-type specific expression in the human brain of *WFS1*-related genes, derived from the BrainCloud database (prefrontal cortex). **d**) Cell-type specific expression in the human brain of *WFS1*-related genes. Gene list derived from BrainSpan database, ages 4 months-4 years. **e**) Key to CSEA map. Hexagon size is scaled to gene list length, and each concentric ring corresponds with specificity index threshold (pSI) which decreases as the number of relatively enriched transcripts decreases and the remaining subset is relatively more specific. Map key reprinted with permission from [68]

To better understand the temporal and spatial expression of *WFS1* in the brain, we examined *WFS1* expression in data from the BrainSpan Atlas of the Developing Human Brain (http://www.brainspan.org), a publicly available human-brain genomic database maintained by the Allen Institute [67]. We constructed heatmaps both before (Fig. 4a, left) and after (Fig. 4a, right) a normalization of gene expression, which was performed by dividing each region’s *WFS1* expression in a certain time period (e.G. *striatum* at 8-15 yrs) by the maximum expression value which that region displays across time periods. The pre-normalisation heatmap enables comparison of relative *WFS1* expression, while the normalized heatmap displays more dynamic range for within-region comparisons. When normalized to maximum signal in each region and classified by age, *WFS1* was found to be most highly expressed in the human brain from 8 to 15 years of age (Fig. 4a), suggesting that *WFS1* may be most active in early brain development rather than in adulthood. Previous studies have indicated that this late childhood and early adolescence period overlaps with a period of active myelination in development. The period of most rapid myelination varies depending on specific cortical and subcortical regions, but dynamic change in myelination has been observed in pre-adolescence and adolescence in the hippocampal formation [69]. Increases in myelination have also been reported in the adolescent time period in motor and cingulate cortices [70], while another study observed myelination during development in frontopolar and visual neocortex but not motor and somatosensory cortices in adolescence [71]. The expression of *WFS1* and correlated gene sets in certain cell populations can also be examined using the Cell-type Specific Expression Analysis (CSEA) tool (http://genetics.wustl.edu/jdlab/csea-tool-2/) [68]. Leveraging gene sets from human genetic data or gene expression specific to human or mouse populations, the CSEA tool suggests neural populations that may be disrupted in specific neurogenetic disorders, and by extension what circuits might be of interest in further mechanistic studies. The CSEA tool uses cell-type specific profiling data to evaluate how disease-related genes and transcripts are enriched in candidate cell populations [72]. To further investigate *WFS1’s* role in healthy human brain development, we therefore compiled data regarding *WFS1* expression patterns by obtaining the top 352 genes co-expressed with *WFS1* in the BrainSpan Atlas of the Developing Human Brain. Many functionally related genes are co-expressed, therefore an examination of genes spatially and temporally expressed with *WFS1* may provide functional insights into *WFS1*’s role in the brain. Co-expression with *WFS1* of each gene in the BrainSpan database was calculated by examining expression levels in 35 human participants (starting as young as 8 weeks post-conception) and in each of their brain regions for which gene expression data was available. For age-specific analyses, only the subset of samples which originated from individuals within the age parameters was analyzed. The Pearson correlation coefficient between *WFS1* and each gene’s overall expression across all samples was then calculated and ranked to select the group of genes most highly co-expressed with *WFS1* for further analysis. To replicate this pattern, we also queried the top 304 genes co-expressed with *WFS1* in the BrainCloud application (http://braincloud.jhmi.edu/), which provides a database of gene expression data in the human prefrontal cortex from 14 gestational weeks to 78 years of age [73].

Using CSEA to evaluate the 296 genes in our Brainspan dataset that existed in the cell-type expression dataset, we identified that *WFS1*-correlated genes are enriched in cell populations that include astrocytes and oligodendrocytes in both the cortex and cerebellum, as well as Bergmann glia and oligodendrocytes in the cerebellum (Fig. 4b). Interestingly for our development hypothesis, enrichment also seemed to occur in oligodendrocyte progenitors. Analysis of the 224 genes from our BrainCloud-derived *WFS1*-related gene set that existed in the CSEA expression dataset corroborated the finding of cell-type specific enrichment in oligodendrocyte and astrocyte populations, as well as cerebellar Bergmann glia and oligodendrocytes (Fig. 4c; Additional file 1). Surprisingly, inclusion of astrocytes as a cell type of interest also raises the possibility that astrocytic ER stress or glial-wide impairment may also play a role in myelination alterations and brain changes observed in Wolfram syndrome. However, the exact role of astrocytes in *WFS1*-related disease has not been studied previously and is beyond the scope of this review.

Age-specific analysis of *WFS1*-related gene expression in the 4 months to 4 year time period overlapping with the myelination window also suggested a strong link to glial processes in both CSEA and gene ontology findings. When gene expression was broken down to look at cell-type specific expression across age (Additional file 2), CSEA analysis of the 280 *WFS1*-related genes in the CSEA dataset and derived from the BrainSpan database also revealed enriched expression in glial cells and oligodendrocyte progenitors in the 4 month to 4 year range (Fig. 4d), again suggesting that *WFS1* is implicated in development and preferentially expressed in glia including oligodendrocytes. Gene ontology (GO) analysis of this 4 month-4 year *WFS1*-related gene set using BiNGO, a biological network gene ontology tool (https://www.psb.ugent.be/cbd/papers/BiNGO/Home.html) [74], and the EBI Gene Ontology Annotation Database (2019-03-18 release) [75, 76], also recovered oligodendrocyte- and glia-related terms such as oligodendrocyte differentiation, axon ensheathment, ensheathment of neurons, myelination, gliogenesis, glial cell differentiation, oligodendrocyte development, and glial cell development which were within the top 10 most statistically overrepresented biological processes at this age (*p* < 9E-11, FDR corrected, Additional file 3). Taken together, these gene expression data suggest a role for *WFS1* in myelination by the gene’s co-expression with transcripts characteristic of these cell types and by the gene’s peak expression during developmental periods related to glial maturation. While this co-expression analysis is intriguing, careful work in genetically tractable experimental systems will be needed to determine if *WFS1* mutation is acting directly in glia or indirectly in a non-cell autonomous manner on these maturation processes.

## Neuroimaging in other CNS disorders compared to Wolfram syndrome

Given our neuroimaging findings, suggestions from neuropathological case studies, and our *WFS1* expression analyses, we propose that Wolfram syndrome could be classified as a developmental hypomyelinating condition, characterized by reduced or absent myelin development [77]. As mentioned in the previous section, this group of disorders includes PMD and VWMD. The quantitative neuroimaging findings in PMD and VWM resemble the findings observed in Wolfram syndrome [78, 79]. For example, DTI studies of PMD show widespread decrease in FA and increase in RD, just as we see in Wolfram syndrome [78]. In addition, these hypomyelinating conditions also have cerebellar atrophy, signal abnormalities in the pons, and T2 lesions in the deep white matter [79]. Nevertheless, the signal intensity abnormalities (presumably reflecting defective myelination) observed in PMD and VWM are more extensive, often symmetrical, and appear earlier in life compared with the changes observed in Wolfram syndrome. Furthermore, the clinical course in hypomyelinating condition, unlike Wolfram syndrome, is more precipitous with progressive deterioration of cognitive and motor function occurring in the first and second decades of life. Assessment of myelin deficit in Wolfram syndrome using advanced techniques like magnetization transfer imaging (MTI) and myelin water fraction imaging could elucidate additional information about the specific myelin alterations associated with Wolfram syndrome.

The brain atrophy and the changes in signal intensity patterns in Wolfram syndrome also resemble the patterns observed in another group of rare disorders involving the pontocerebellar tract, e.g., olivopontocerebellar atrophy (OPCA), multiple system atrophy (MSA), and familial spinocerebellar degeneration [14, 43, 80–82]. Specifically, both Wolfram syndrome and OPCA show atrophy involving the cerebellum, pons, and middle cerebellar peduncles with relative sparing of the pyramidal tracts [82]. However, the severity and chronology of the clinical manifestations can be widely different between these conditions. For example, the age of onset in sporadic cases of OPCA and MSA is usually in the fifth or sixth decade, while the neurological manifestations in Wolfram syndrome can be evident in the second decade. The preferential involvement of the middle cerebellar peduncle is a striking shared feature between OPCA and Wolfram syndrome. It is intriguing to know that the cerebellum and brainstem have a neurobiologically-linked course of development with preferential susceptibility to neurodevelopmental disorders [83, 84]. Besides, this specific pattern of degeneration and neuronal loss in the basilar part of the pons, cerebellum, and inferior olivary nuclei is observed in several neurodegenerative disorders like mitochondrial disease and olivopontocerebellar atrophy [85]. Several brainstem nuclei are closely connected with the cerebellum and lesions in either one can result in a degeneration of the other. Why these structures are especially vulnerable in Wolfram syndrome is yet to be determined.

## Conclusions

In summary, Wolfram syndrome is a neurological disorder with features of abnormal brain development and neurodegeneration. Emerging evidence from neuroimaging and molecular genetic studies indicates abnormal myelination and oligodendrocyte dysfunction are important features of the disease. Studies of *WFS1* expression and function in oligodendrocytes and glial cells are limited and could be used to test our hypothesis. Despite the advances in describing the gross neurologic alterations in animal models of Wolfram syndrome [86], animal models need to have a well-described neurophenotype that parallels what is seen in humans, particularly the developmental aspects. Another avenue to explore could be the study of human induced pluripotent stem cells (hiPSCs)-derived oligodendrocytes from individuals with Wolfram syndrome, e.g., using oligocortical spheroids [87, 88]. This information could help us understand the geno-phenotype relationship in Wolfram syndrome, identify myelin-related biological markers for disease progression and treatment response, and open the possibility to look into remyelination therapies as a potential intervention to stop neurological deterioration in Wolfram syndrome [76]. Furthermore, understanding how Wolfram syndrome affects brain structure and function could also help identify potential connections between neurodevelopmental disorders and neurodegeneration.

## Supplementary information

**Additional file 1. **A list of the most highly correlated genes with *WFS1* in BrainCloud database, available in CSEA dataset.

**Additional file 2. **Cell-type specific expression of *WFS1* related genes in the CSEA dataset and derived from the BrainSpan database across several age groups.

**Additional file 3. **A list of biological processes from Gene ontology analysis of 4 month-4 years *WFS1*-related gene set using BiNGO, a biological network gene ontology tool.

## Data Availability

The datasets used and/or analyzed during the current study are available from the corresponding author on reasonable request.

## Abbreviations

AD: Axial diffusivity
ALIC: Anterior limb of internal capsule
AR: Acoustic radiation
ATF6: Activating transcription factor 6
BiNGO: Biological network gene ontology tool
Ca^2+^: Calcium
CC: Corpus callosum
CHD: Congenital heart disease
CNS: Central nervous system
CSEA: Cell-type specific expression analysis
CST: Corticospinal tract
CST: Corticospinal tract
DI: Diabetes insipidus
DIDMOAD: Diabetes insipidus diabetes mellitus optic atrophy and deafness
DM: Diabetes mellitus
DTI: Diffusion tensor imaging
ER: Endoplasmic reticulum
ERIS: ER intermembrane small protein
FA: Fractional anisotropy
FDR: False discovery rate
FLAIR: Fluid attenuation inversion recovery
GM: Grey matter
IFOF: Inferior fronto-occipital fasciculus
ILF: Inferior longitudinal fasciculus
ION: Inferior olivary nucleus
LGN: Lateral geniculate nucleus
MAMs: Mitochondria-associated ER membranes
MCP: Middle cerebellar peduncle
MD: Mean diffusivity
MRI: Magnetic resonance imaging
MSA: Multiple system atrophy
OA: Optic atrophy
OPCA: Olivopontocerebellar atrophy
OPCT: Olivopontocerebellar tract
OR: Optic radiation
pcw: Postconception week
PET-CT: Positron emission tomography – computed tomography
PMD: Pelizaeus-Merzbacher disease
pSI: Specificity index threshold
PVN: Paraventricular nucleus of hypothalamus
QDEC: Query, design, estimate, contrast
RD: Radial diffusivity
ROI: Region of interest
SLF: Superior longitudinal fasciculus
SN: Substantia nigra
SNHL: Sensory neural hearing loss
SON: Supraoptic nucleus
TBSS: Tract-based spatial statistics
UPR: Unfolded protein response
VBM: Voxel-based morphometry
VWMD: Vanishing white matter disease
WM: White matter

## References

[1] Barrett TG, Bundey SE, Macleod AF (1995). Neurodegeneration and diabetes: UK nationwide study of Wolfram (DIDMOAD) syndrome. Lancet.

[2] Doty T, Foster ER, Marshall B, Ranck S, Hershey T (2017). The effects of disease-related symptoms on daily function in Wolfram syndrome. Transl Sci Rare Dis.

[3] de Heredia ML, Cleries R, Nunes V (2013). Genotypic classification of patients with Wolfram syndrome: insights into the natural history of the disease and correlation with phenotype. Genet Med.

[4] Amr S, Heisey C, Zhang M, Xia XJ, Shows KH, Ajlouni K (2007). A homozygous mutation in a novel zinc-finger protein, ERIS, is responsible for Wolfram syndrome 2. Am J Hum Genet.

[5] Inoue H, Tanizawa Y, Wasson J, Behn P, Kalidas K, Bernal-Mizrachi E (1998). A gene encoding a transmembrane protein is mutated in patients with diabetes mellitus and optic atrophy (Wolfram syndrome). Nat Genet.

[6] al-Sheyyab M, Jarrah N, Younis E, Shennak MM, Hadidi A, Awidi A (2001). Bleeding tendency in Wolfram syndrome: a newly identified feature with phenotype genotype correlation. Eur J Pediatr.

[7] Bai X, Lv H, Zhang F, Liu J, Fan Z, Xu L (2014). Identification of a novel missense mutation in the WFS1 gene as a cause of autosomal dominant nonsyndromic sensorineural hearing loss in all-frequencies. Am J Med Genet A.

[8] Eiberg H, Hansen L, Kjer B, Hansen T, Pedersen O, Bille M (2006). Autosomal dominant optic atrophy associated with hearing impairment and impaired glucose regulation caused by a missense mutation in the WFS1 gene. J Med Genet.

[9] Zalloua PA, Azar ST, Delepine M, Makhoul NJ, Blanc H, Sanyoura M (2008). WFS1 mutations are frequent monogenic causes of juvenile-onset diabetes mellitus in Lebanon. Hum Mol Genet.

[10] Takeda K, Inoue H, Tanizawa Y, Matsuzaki Y, Oba J, Watanabe Y (2001). WFS1 (Wolfram syndrome 1) gene product: predominant subcellular localization to endoplasmic reticulum in cultured cells and neuronal expression in rat brain. Hum Mol Genet.

[11] Takei D, Ishihara H, Yamaguchi S, Yamada T, Tamura A, Katagiri H (2006). WFS1 protein modulates the free Ca (2+) concentration in the endoplasmic reticulum. FEBS Lett.

[12] Altpere A, Raud S, Sutt S, Reimets R, Visnapuu T, Toots M (2018). Mild stress induces brain region-specific alterations of selective ER stress markers' mRNA expression in Wfs1-deficient mice. Behav Brain Res.

[13] Bonnet Wersinger Delphine, Benkafadar Nesrine, Jagodzinska Jolanta, Hamel Christian, Tanizawa Yukio, Lenaers Guy, Delettre Cécile (2014). Impairment of Visual Function and Retinal ER Stress Activation in Wfs1-Deficient Mice. PLoS ONE.

[14] Leiva-Santana C, Carro-Martinez A, Monge-Argiles A, Palao-Sanchez A (1993). Neurologic manifestations in Wolfram’s syndrome. Rev Neurol.

[15] Scolding NJ, Kellar-Wood HF, Shaw C, Shneerson JM, Antoun N (1996). Wolfram syndrome: hereditary diabetes mellitus with brainstem and optic atrophy. Ann Neurol.

[16] Hershey T, Lugar HM, Shimony JS, Rutlin J, Koller JM, Perantie DC (2012). Early brain vulnerability in Wolfram syndrome. PLoS One.

[17] Pickett KA, Duncan RP, Hoekel J, Marshall B, Hershey T, Earhart GM (2012). Early presentation of gait impairment in Wolfram syndrome. Orphanet J Rare Dis..

[18] Nickl-Jockschat T, Kunert HJ, Herpertz-Dahlmann B, Grozinger M (2008). Psychiatric symptoms in a patient with Wolfram syndrome caused by a combination of thalamic deficit and endocrinological pathologies. Neurocase..

[19] Bischoff AN, Reiersen AM, Buttlaire A, Al-Lozi A, Doty T, Marshall BA (2015). Selective cognitive and psychiatric manifestations in Wolfram syndrome. Orphanet J Rare Dis.

[20] Luuk H, Koks S, Plaas M, Hannibal J, Rehfeld JF, Vasar E (2008). Distribution of Wfs1 protein in the central nervous system of the mouse and its relation to clinical symptoms of the Wolfram syndrome. J Comp Neurol.

[21] Visnapuu T, Plaas M, Reimets R, Raud S, Terasmaa A, Koks S (2013). Evidence for impaired function of dopaminergic system in Wfs1-deficient mice. Behav Brain Res.

[22] Sakakibara Y, Sekiya M, Fujisaki N, Quan X, Iijima KM (2018). Knockdown of wfs1, a fly homolog of Wolfram syndrome 1, in the nervous system increases susceptibility to age- and stress-induced neuronal dysfunction and degeneration in Drosophila. PLoS Genet.

[23] Morikawa S, Tajima T, Nakamura A, Ishizu K, Ariga T (2017). A novel heterozygous mutation of the WFS1 gene leading to constitutive endoplasmic reticulum stress is the cause of Wolfram syndrome. Pediatr Diabetes.

[24] Rouzier C, Moore D, Delorme C, Lacas-Gervais S, Ait-El-Mkadem S, Fragaki K (2017). A novel CISD2 mutation associated with a classical Wolfram syndrome phenotype alters Ca2+ homeostasis and ER-mitochondria interactions. Hum Mol Genet.

[25] Lu S, Kanekura K, Hara T, Mahadevan J, Spears LD, Oslowski CM (2014). A calcium-dependent protease as a potential therapeutic target for Wolfram syndrome. Proc Natl Acad Sci U S A.

[26] Hetz C, Saxena S (2017). ER stress and the unfolded protein response in neurodegeneration. Nat Rev Neurol.

[27] Oakes SA, Papa FR (2015). The role of endoplasmic reticulum stress in human pathology. Annu Rev Pathol.

[28] Schroder M, Kaufman RJ (2005). ER stress and the unfolded protein response. Mutat Res.

[29] Urra H, Dufey E, Lisbona F, Rojas-Rivera D, Hetz C (2013). When ER stress reaches a dead end. Biochim Biophys Acta.

[30] Fonseca SG, Ishigaki S, Oslowski CM, Lu S, Lipson KL, Ghosh R (2010). Wolfram syndrome 1 gene negatively regulates ER stress signaling in rodent and human cells. J Clin Invest.

[31] Chen YF, Wu CY, Kirby R, Kao CH, Tsai TF. A role for the CISD2 gene in lifespan control and human disease. Ann N Y Acad Sci. 2010. 10.1111/j.1749-6632.2010.05619.x 20649540

[32] Ross-Cisneros FN, Pan BX, Silva RA, Miller NR, Albini TA, Tranebjaerg L (2013). Optic nerve histopathology in a case of Wolfram syndrome: a mitochondrial pattern of axonal loss. Mitochondrion.

[33] Kanki T, Klionsky DJ (2009). Mitochondrial abnormalities drive cell death in Wolfram syndrome 2. Cell Res.

[34] Cagalinec M, Liiv M, Hodurova Z, Hickey MA, Vaarmann A, Mandel M (2016). Role of mitochondrial dynamics in neuronal development: mechanism for wolfram syndrome. PLoS Biol.

[35] Angebault Claire, Fauconnier Jérémy, Patergnani Simone, Rieusset Jennifer, Danese Alberto, Affortit Corentin A., Jagodzinska Jolanta, Mégy Camille, Quiles Mélanie, Cazevieille Chantal, Korchagina Julia, Bonnet-Wersinger Delphine, Milea Dan, Hamel Christian, Pinton Paolo, Thiry Marc, Lacampagne Alain, Delprat Benjamin, Delettre Cécile (2018). ER-mitochondria cross-talk is regulated by the Ca2+ sensor NCS1 and is impaired in Wolfram syndrome. Science Signaling.

[36] Marchi S, Patergnani S, Pinton P (2014). The endoplasmic reticulum-mitochondria connection: one touch, multiple functions. Biochim Biophys Acta.

[37] Carreras-Sureda A, Pihan P, Hetz C (2017). The unfolded protein response: at the intersection between endoplasmic reticulum function and mitochondrial bioenergetics. Front Oncol.

[38] Carson MJ, Slager UT, Steinberg RM (1977). Simultaneous occurrence of diabetes mellitus, diabetes insipidus, and optic atrophy in a brother and sister. Am J Dis Child.

[39] Genis D, Davalos A, Molins A, Ferrer I (1997). Wolfram syndrome: a neuropathological study. Acta Neuropathol.

[40] Hilson JB, Merchant SN, Adams JC, Joseph JT (2009). Wolfram syndrome: a clinicopathologic correlation. Acta Neuropathol.

[41] Shannon P, Becker L, Deck J (1999). Evidence of widespread axonal pathology in Wolfram syndrome. Acta Neuropathol.

[42] Lugar HM, Koller JM, Rutlin J, Eisenstein SA, Neyman O, Narayanan A (2019). Evidence for altered neurodevelopment and neurodegeneration in Wolfram syndrome using longitudinal morphometry. Sci Rep.

[43] Ito S, Sakakibara R, Hattori T (2007). Wolfram syndrome presenting marked brain MR imaging abnormalities with few neurologic abnormalities. AJNR Am J Neuroradiol.

[44] Gocmen R, Guler E (2014). Teaching NeuroImages: MRI of brain findings of Wolfram (DIDMOAD) syndrome. Neurology.

[45] Galluzzi P, Filosomi G, Vallone IM, Bardelli AM, Venturi C (1999). MRI of Wolfram syndrome (DIDMOAD). Neuroradiology.

[46] Harsha KJ, Parameswaran K (2016). Wolfram (DIDMOAD) syndrome with ventral central pontine hyperintensity without brainstem atrophy. Neurol India.

[47] Pakdemirli E, Karabulut N, Bir LS, Sermez Y (2005). Cranial magnetic resonance imaging of Wolfram (DIDMOAD) syndrome. Australas Radiol.

[48] Elli FM, Ghirardello S, Giavoli C, Gangi S, Dioni L, Crippa M (2012). A new structural rearrangement associated to Wolfram syndrome in a child with a partial phenotype. Gene.

[49] Lugar HM, Koller JM, Rutlin J, Marshall BA, Kanekura K, Urano F (2016). Neuroimaging evidence of deficient axon myelination in Wolfram syndrome. Sci Rep.

[50] Alexander AL, Lee JE, Lazar M, Field AS (2007). Diffusion tensor imaging of the brain. Neurotherapeutics.

[51] Song SK, Sun SW, Ramsbottom MJ, Chang C, Russell J, Cross AH (2002). Dysmyelination revealed through MRI as increased radial (but unchanged axial) diffusion of water. Neuroimage.

[52] Zmyslowska A, Malkowski B, Fendler W, Borowiec M, Antosik K, Gnys P (2014). Central nervous system PET-CT imaging reveals regional impairments in pediatric patients with Wolfram syndrome. PLoS One.

[53] Ghirardello S, Dusi E, Castiglione B, Fumagalli M, Mosca F (2014). Congenital central diabetes insipidus and optic atrophy in a Wolfram newborn: is there a role for WFS1 gene in neurodevelopment?. Ital J Pediatr.

[54] Hadidy AM, Jarrah NS, Al-Till MI, El-Shanti HE, Ajlouni KM (2004). Radiological findings in Wolfram syndrome. Saudi Med J.

[55] Ivask M, Hugill A, Koks S (2016). RNA-sequencing of WFS1-deficient pancreatic islets. Physiol Rep.

[56] Godin JD, Creppe C, Laguesse S, Nguyen L (2016). Emerging roles for the unfolded protein response in the developing nervous system. Trends Neurosci.

[57] Khacho M, Slack RS (2018). Mitochondrial dynamics in the regulation of neurogenesis: from development to the adult brain. Dev Dyn.

[58] Roboti P, Swanton E, High S (2009). Differences in endoplasmic-reticulum quality control determine the cellular response to disease-associated mutants of proteolipid protein. J Cell Sci.

[59] Southwood CM, Garbern J, Jiang W, Gow A (2002). The unfolded protein response modulates disease severity in Pelizaeus-Merzbacher disease. Neuron.

[60] Popko B (2010). Myelin maintenance: axonal support required. Nat Neurosci.

[61] Kawano J, Fujinaga R, Yamamoto-Hanada K, Oka Y, Tanizawa Y, Shinoda K (2009). Wolfram syndrome 1 (Wfs1) mRNA expression in the normal mouse brain during postnatal development. Neurosci Res.

[62] Kawano J, Tanizawa Y, Shinoda K (2008). Wolfram syndrome 1 (Wfs1) gene expression in the normal mouse visual system. J Comp Neurol.

[63] Yurimoto S, Hatano N, Tsuchiya M, Kato K, Fujimoto T, Masaki T (2009). Identification and characterization of wolframin, the product of the wolfram syndrome gene (WFS1), as a novel calmodulin-binding protein. Biochemistry.

[64] Simons Mikael, Nave Klaus-Armin (2015). Oligodendrocytes: Myelination and Axonal Support. Cold Spring Harbor Perspectives in Biology.

[65] Bauer J, Bradl M, Klein M, Leisser M, Deckwerth TL, Wekerle H (2002). Endoplasmic reticulum stress in PLP-overexpressing transgenic rats: gray matter oligodendrocytes are more vulnerable than white matter oligodendrocytes. J Neuropathol Exp Neurol.

[66] Lin W, Popko B (2009). Endoplasmic reticulum stress in disorders of myelinating cells. Nat Neurosci.

[67] Miller JA, Ding SL, Sunkin SM, Smith KA, Ng L, Szafer A (2014). Transcriptional landscape of the prenatal human brain. Nature.

[68] Xu X, Wells AB, O'Brien DR, Nehorai A, Dougherty JD (2014). Cell type-specific expression analysis to identify putative cellular mechanisms for neurogenetic disorders. J Neurosci.

[69] Benes FM, Turtle M, Khan Y, Farol P (1994). Myelination of a key relay zone in the hippocampal formation occurs in the human brain during childhood, adolescence, and adulthood. Arch Gen Psychiatry.

[70] Kwon D, Pfefferbaum A, Sullivan EV, Pohl KM (2018). Regional growth trajectories of cortical myelination in adolescents and young adults: longitudinal validation and functional correlates. Brain Imaging Behav.

[71] Miller DJ, Duka T, Stimpson CD, Schapiro SJ, Baze WB, McArthur MJ (2012). Prolonged myelination in human neocortical evolution. Proc Natl Acad Sci U S A.

[72] Doyle JP, Dougherty JD, Heiman M, Schmidt EF, Stevens TR, Ma G (2008). Application of a translational profiling approach for the comparative analysis of CNS cell types. Cell.

[73] Colantuoni C, Lipska BK, Ye T, Hyde TM, Tao R, Leek JT (2011). Temporal dynamics and genetic control of transcription in the human prefrontal cortex. Nature.

[74] Maere S, Heymans K, Kuiper M (2005). BiNGO: a Cytoscape plugin to assess overrepresentation of gene ontology categories in biological networks. Bioinformatics.

[75] Ashburner M, Ball CA, Blake JA, Botstein D, Butler H, Cherry JM (2000). Gene ontology: tool for the unification of biology. The Gene Ontology Consortium. Nat Genet.

[76] Kremer D, Akkermann R, Kury P, Dutta R (2019). Current advancements in promoting remyelination in multiple sclerosis. Mult Scler.

[77] Barkovich AJ, Deon S (2016). Hypomyelinating disorders: an MRI approach. Neurobiol Dis.

[78] Laukka JJ, Makki MI, Lafleur T, Stanley J, Kamholz J, Garbern JY (2014). Diffusion tensor imaging of patients with proteolipid protein 1 gene mutations. J Neurosci Res.

[79] Steenweg ME, Vanderver A, Blaser S, Bizzi A, de Koning TJ, Mancini GM (2010). Magnetic resonance imaging pattern recognition in hypomyelinating disorders. Brain.

[80] Burk K, Abele M, Fetter M, Dichgans J, Skalej M, Laccone F (1996). Autosomal dominant cerebellar ataxia type I clinical features and MRI in families with SCA1, SCA2 and SCA3. Brain.

[81] Gilman S, Sima AA, Junck L, Kluin KJ, Koeppe RA, Lohman ME (1996). Spinocerebellar ataxia type 1 with multiple system degeneration and glial cytoplasmic inclusions. Ann Neurol.

[82] Savoiardo M, Strada L, Girotti F, Zimmerman RA, Grisoli M, Testa D (1990). Olivopontocerebellar atrophy: MR diagnosis and relationship to multisystem atrophy. Radiology.

[83] Limperopoulos C, du Plessis AJ (2006). Disorders of cerebellar growth and development. Curr Opin Pediatr.

[84] Barkovich AJ, Millen KJ, Dobyns WB (2009). A developmental and genetic classification for midbrain-hindbrain malformations. Brain.

[85] Roubertie A, Leboucq N, Picot MC, Nogue E, Brunel H, Le Bars E (2015). Neuroradiological findings expand the phenotype of OPA1-related mitochondrial dysfunction. J Neurol Sci.

[86] Plaas M, Seppa K, Reimets R, Jagomae T, Toots M, Koppel T (2017). Wfs1- deficient rats develop primary symptoms of Wolfram syndrome: insulin-dependent diabetes, optic nerve atrophy and medullary degeneration. Sci Rep.

[87] Madhavan M, Nevin ZS, Shick HE, Garrison E, Clarkson-Paredes C, Karl M (2018). Induction of myelinating oligodendrocytes in human cortical spheroids. Nat Methods.

[88] Nevin ZS, Factor DC, Karl RT, Douvaras P, Laukka J, Windrem MS (2017). Modeling the mutational and phenotypic landscapes of Pelizaeus-Merzbacher disease with human iPSC-derived Oligodendrocytes. Am J Hum Genet.

