# Supplementary figures and images for: Developmental hypomyelination in Wolfram syndrome: new insights from neuroimaging and gene expression analyses
Source: Orphanet J Rare Dis. 2019 Dec 3;14:279. doi: 10.1186/s13023-019-1260-9 (PMC6889680; doi:10.1186/s13023-019-1260-9)

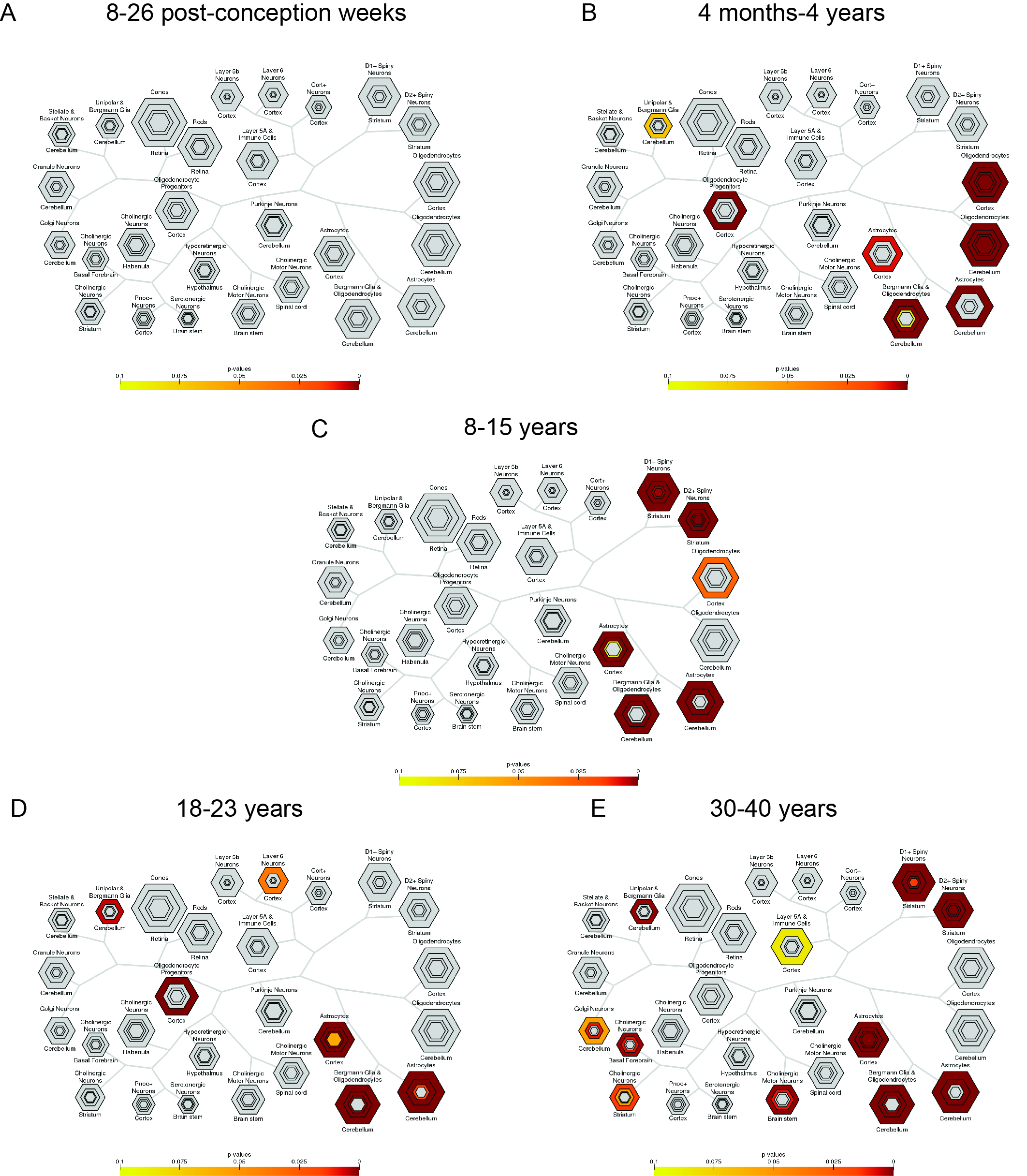

Supplement: Supplementary file 2 — Additional file 2. Cell-type specific expression of WFS1 related genes in the CSEA dataset and derived from the BrainSpan database across several age groups. [file 13023_2019_1260_MOESM2_ESM.jpg]
